# Sequential transplantation of exosomes and mesenchymal stem cells pretreated with a combination of hypoxia and Tongxinluo efficiently facilitates cardiac repair

**DOI:** 10.1186/s13287-022-02736-z

**Published:** 2022-02-07

**Authors:** Yuyan Xiong, Ruijie Tang, Junyan Xu, Wenyang Jiang, Zhaoting Gong, Lili Zhang, Xiaosong Li, Yu Ning, Peisen Huang, Jun Xu, Guihao Chen, Chen Jin, Xiangdong Li, Haiyan Qian, Yuejin Yang

**Affiliations:** State Key Laboratory of Cardiovascular Disease, Department of Cardiology, Fuwai Hospital, National Center for Cardiovascular Diseases, Chinese Academy of Medical Science and Peking Union Medical College, Beijing, 100037 China

**Keywords:** Exosome, MSC, SDF-1, CXCR4, Myocardial infarction

## Abstract

**Background:**

Bone marrow-derived mesenchymal stem cells (MSCs), which possess immunomodulatory characteristic, are promising candidates for the treatment of acute myocardial infarction (AMI). However, the low retention and survival rate of MSCs in the ischemic heart limit their therapeutic efficacy. Strategies either modifying MSCs or alleviating the inflammatory environment, which facilitates the recruitment and survival of the engrafted MSCs, may solve the problem. Thus, we aimed to explore the therapeutic efficacy of sequential transplantation of exosomes and combinatorial pretreated MSCs in the treatment of AMI.

**Methods:**

Exosomes derived from MSCs were delivered to infarcted hearts through intramyocardial injection followed by the intravenous infusion of differentially pretreated MSCs on Day 3 post-AMI. Enzyme linked immunosorbent assay (ELISA) was performed to evaluate the inflammation level as well as the SDF-1 levels in the infarcted border zone of the heart. Echocardiography and histological analysis were performed to assess cardiac function, infarct size, collagen area and angiogenesis.

**Results:**

Sequential transplantation of exosomes and the combinatorial pretreated MSCs significantly facilitated cardiac repair compared to AMI rats treated with exosomes alone. Notably, compared to the other three methods of cotransplantation, combinatorial pretreatment with hypoxia and Tongxinluo (TXL) markedly enhanced the CXCR4 level of MSCs and promoted recruitment, which resulted in better cardiac function, smaller infarct size and enhanced angiogenesis. We further demonstrated that exosomes effectively reduced apoptosis in MSCs in vitro.

**Conclusion:**

Sequential delivery of exosomes and pretreated MSCs facilitated cardiac repair post-AMI, and combined pretreatment with hypoxia and TXL better enhanced the cardioprotective effects. This method provides new insight into the clinical translation of stem cell-based therapy for AMI.

## Background

Cardiovascular disease (CVD), including acute myocardial infarction (AMI), remains the leading cause of morbidity and mortality worldwide [1–3]. Myocardial infarction injury triggers sudden extensive cardiomyocytes loss accompanied by an intense inflammatory response, which might lead to deteriorated cardiac function, post-infarction remodeling and heart failure [4, 5]. Stem cell-based therapy is considered a promising strategy for facilitating cardiac repair [6–8], and bone marrow-derived mesenchymal stem cells (MSCs) are ideal candidates due to their immunomodulatory characteristics [9–11]. However, the low retention and poor survival of the transplanted MSCs in the ischemic heart limit their therapeutic efficacy [12, 13]. Thus, methods either modifying MSCs, or alleviating the inflammatory environment which facilitates the recruitment and survival of MSCs, may solve the problem [14–16].

The stromal cell-derived factor 1 (SDF-1)/CXC chemokine receptor 4 (CXCR4) axis plays pivotal role in facilitating cardiac repair and the recruitment of stem cells to the infarcted myocardium after ischemic injury [17–23]. Myocardial expression of SDF-1 has been confirmed to peak in the early phase of AMI and decrease to a relatively low level in the following days [17, 24, 25]. Therefore, transplantation of MSCs in the early phase may achieve a higher retention rate. However, during the same early stage of AMI, an intense inflammation response occurs and enormous amounts of inflammatory cytokines are released, which is disastrous for transplanted cell survival. Elevating SDF-1 levels while limiting inflammation levels might promote the recruitment and survival of the transplanted cells, thus improving their therapeutic efficacy.

Exosomes, 30–150 nm extracellular vesicles, contain various bioactive proteins and RNAs, and are vital in intercellular communication [26, 27]. It has been widely reported to exhibit distinct benefits in promoting cardiac recovery via augmenting cell survival [28–31], ameliorating inflammation [32–34], and increasing the myocardial SDF-1 expression level [35] at the early stage in the infarcted heart. In addition, numerous strategies including drug pretreatment [36, 37], hypoxia preconditioning [38, 39], genetic modifications [40–42] and tissue engineering [43, 44] have been developed to augment the therapeutic efficacy of MSCs. Among these methods, drug pretreatment such as with stains and Tongxinluo (TXL), as well as hypoxic preconditioning are widely used, which also have potential for clinical translation. TXL, a traditional Chinese medicine compound, is capable of protecting MSCs against hypoxic injury [45]. Additionally, hypoxia preconditioning could augment the therapeutic efficacy of MSCs in myocardial injury treatment [39, 46]. Whether a combined strategy of hypoxia and TXL pretreatment could better enhance the therapeutic efficacy of MSCs has not been explored yet.

In the present study, we first explored the cardioprotective effects of sequential delivery of exosomes and the combinatorially pretreated MSCs in the treatment of AMI, and further investigated the effects of combined pretreatment with hypoxia and TXL of MSCs. Exosome transplantation in the early stage of AMI ameliorated inflammation, reduced apoptosis and increased myocardial SDF-1, thus creating a better microenvironment for the survival and recruitment of MSCs. Meanwhile, combined pretreatment with hypoxia and TXL enhanced the expression level of CXCR4 and increased the retention rate of MSCs. Sequential transplantation of exosomes and MSCs pretreated with both hypoxia and TXL resulted in better cardiac recovery. Thus, our data suggested that sequential transplantation of exosomes and the pretreated MSCs significantly facilitated cardiac repair, and a combination pretreatment with hypoxia and TXL further augmented the therapeutic efficacy of MSCs, providing novel insights for the clinical treatment of AMI.

## Methods

### Animals

All animal experiments conformed to the guidelines for animal care and were approved by the Institutional Animal Care and Use Committee of Fuwai Hospital, Chinese Academy of Medical Sciences and Peking Union Medical College.

### MSCs isolation and culture

MSCs were isolated from femoral and tibial bone marrow of male Sprague–Dawley (SD) rats (60–80 g) and cultured in a humidified incubator. Briefly, bone marrow was flushed with Iscove’s modified Dulbecco’s medium (IMDM, Invitrogen, USA) supplemented with 10% fetal bovine serum (FBS, Gibco, USA) and 1% penicillin/streptomycin (Gibco, USA). Flow cytometry was employed to identify MSCs at passage 3 with antibodies against CD90 (eBioscience, USA), CD29 (eBioscience, USA), CD45 (eBioscience, USA), CD11 (BD Bioscience, USA). MSCs at passage 3, 4 were used for experiments.

### Pretreatment of MSCs

Ultrafine TXL powder (Shijiazhuang Yiling Pharmaceutical Co., Shijiazhuang, China) was dissolved in serum-free IMDM, and then the suspension was sonicated and centrifuged. Sterile TXL solution was obtained by filtering the supernatant through a 0.22-μm filter. The solution was adjusted to a final concentration of 2 mg/mL by adding IMDM and then stored at 4 °C or − 20 °C until use. For TXL pretreatment (MSC^T^), when passage 3–4 MSCs grew to approximately 60%, 400 μg/mL TXL was added to the medium for 24 h. For hypoxia pretreatment (MSC^H^), when passage 3–4 MSCs grew to approximately 60%, MSCs were cultured in a humidified incubator with 1% O_2_ at 37 °C for 24 h. For combined pretreatment with TXL and hypoxia (MSC^C^), MSCs treated with 400 μg/mL TXL were cultured in a humidified incubator with 1% O_2_ at 37 °C for 24 h.

### Exosomes isolation, identification and labeling

Exosomes were isolated from the conditioned medium of MSCs by differential centrifugation [47]. Briefly, MSCs at passage 3 or 4 were cultivated in IMDM containing 10% FBS and 1% penicillin/streptomycin. When grown to 80–90% confluence, the medium was changed into fresh serum-free IMDM and MSCs were cultured for 48 h. The conditioned supernatants were collected and centrifuged at 300 g for 10 min and 2000 g for 20 min to eliminate cells and debris. Then, the supernatants were centrifuged at 13,500 g for 30 min to remove macrovesicles. The exosomes pellets were obtained by ultracentrifugation at 120,000 g for 70 min at 4 °C, washed in phosphate-buffered saline (PBS, pH 7.4, Gibco, USA) and collected by a second ultracentrifugation at 120,000 g for 70 min. The exosomes were finally resuspended in PBS and stored at − 80 °C for use.

A microBCA protein assay kit (Thermo Scientific, USA) was utilized to measure the protein concentrations of exosomes. The shapes and sizes of exosomes were determined by transmission electron microscopy (TEM, FEI, Tecnai G2 Spirit BioTwin, USA) and nanoparticle tracking analysis (NTA, PARTICLE METRIX, ZetaVIEW, Germany). Besides, western blotting was used to identify the exosomal markers including Alix (Cell Signaling Technology, USA) and TSG101 (Santa Cruz, USA).

For assessment of uptake and distribution of purified exosomes, exosomes were labeled using PKH26 or PKH67 Fluorescent Cell Linker Kit (Sigma-Aldrich, USA) following the manufacturer’s instructions.

### Rat AMI model induction

All surgeries and relevant analyses were performed in a blinded manner. Female SD rats (200–220 g weight) were anesthetized by intraperitoneal injection of pentobarbital sodium (50 mg/kg) before the surgical procedure. The rats were given ibuprofen at a dose of 30 mg/kg by gavage at least 12 h prior to thoracotomy to alleviate preoperative pain and distress. To induce AMI model, the chest was opened gently by left thoracotomy and rats were subjected to the left anterior descending (LAD) coronary artery ligation by a 6-0 silk polyester. Rats were randomized into following groups: Sham group, AMI group (PBS alone), Exo group (exosome treatment alone), MSC group (MSCs treatment alone), Exo + MSC group (Exo combined with MSCs), Exo + MSC^H^ group (Exo combined with MSC^H^), Exo + MSC^T^ group (Exo combined with MSC^T^) and Exo + MSC^C^ group (Exo combined with MSC^C^). The rats in Sham group only underwent the same procedure without LAD ligation. PBS (100 μL) or exosomes (20 μg, in 100 μL PBS) were injected by a 31-gauge Hamilton syringe at three sites around the peri-infarcted myocardium 30 min after LAD ligation. For the following MSC delivery, 2 × 10^6^ MSC, MSC^H^, MSC^T^, or MSC^C^ were injected into the Exo-treated rats via the tail vein at Day 3 postinfarction. After operation, the incisions were sewn up and disinfected, followed by postoperative analgesia with ibuprofen for 1 week [48].

### Cardiac function by echocardiography

All measurements and analyses were performed by an experienced investigator in a blind manner. Cardiac function at baseline (Day 3 after AMI) and the endpoint (4 weeks after AMI) were evaluated by transthoracic echocardiography using a VisualSonics Vevo 2100 system. Briefly, rats were anesthetized by 2% isoflurane in the induction chamber and then a nose cone was placed to maintain sedation level. Rats were placed on a heating pad, keeping the core body temperature ~ 37 °C and heart rate > 350 b.p.m.. M-mode was then used to measure the left ventricular wall thickness and left ventricular inner diameter in systole and diastole. The levels of the left ventricular ejection fraction (LVEF), left ventricular fractional shortening (LVFS), left ventricular end-diastolic volume (LVEDV) and left ventricular end-systolic volume (LVESV) at baseline and the endpoint were measured to assess cardiac function.

### Histological analysis

Four weeks after infarction, rats were euthanized after echocardiography to harvest the hearts. To determine the infarct size and collagen area, hearts were fixed in 4% paraformaldehyde for 72 h, dehydrated and embedded in paraffin. The heart was cut 1 mm below the ligation point perpendicular to the axis of the LAD. Masson trichrome and Sirius red staining were utilized to measure the infarct size and collagen area respectively. The infarct size was calculated as [(epicardial infarct ratio + endocardial infarct ratio)/2] × 100. The epicardial infarct ratio was obtained by dividing the epicardial infarct length by the epicardial circumferences. The endocardial infarct ratio was calculated similarly. Percent area of collagen was calculated as the average ratio of the collagen area to the total area of LV (collagen area/total LV area × 100%).

### Terminal deoxynucleotidyl transferase-mediated dUTP nick-end labeling (TUNEL) assay

An In Situ Cell Death Detection kit (Roche, Germany) was used to assess apoptotic cells in the ischemic myocardium following the manufacturer’s protocols. Briefly, the sections were blocked and incubated with TUNEL reaction mixture for 1 h at room temperature. Then the sections were stained with cardiac troponin T (cTNT, Abcam, 1:200, USA) at 4 °C overnight, and nuclei were stained with DAPI. The stained sections were examined under a confocal microscope at 400× magnification in four randomly chosen fields. Normal nuclei are presented in blue color, and apoptotic nuclei are presented in green. The apoptotic ratio is described as the percentage of apoptotic cells among all cells.

### Immunofluorescence staining

For immunofluorescence staining, the paraffin sections were incubated with primary antibodies at 4 °C overnight followed by incubation with goat anti-mouse (Thermo Fisher Scientific, 1:200, USA) or goat anti-rabbit (Thermo Fisher Scientific, 1:200, USA) highly cross-adsorbed Alexa Fluor 488 or 594 secondary antibodies at room temperature for 1 h. After washed by PBS, sections were stained with DAPI and then observed under a laser scanning confocal microscope (Leica, Germany). Five high-power fields (HPF) per tissue were randomly selected for analysis. Arteriole density and vascular density were reflected by α-smooth muscle actin (α-SMA, Abcam, 1:200, USA) and CD31 (Abcam, 1:300, USA) staining respectively. Arteriole density or vascular density was calculated as the number of α-SMA^+^ cells or CD31^+^ cells per HPF.

### Enzyme linked immunosorbent assay (ELISA) analyses

Rat SDF-1 ELISA kit (elabscience, China, E-EL-R0922c), rat tumor necrosis factor (TNF)- α and rat interleukin (IL)-6 ELISA kits (eBioscience, USA, BMS625 and BMS622) were used to quantify the expression level of SDF-1 and inflammatory cytokines TNF- α and IL-6 in the peri-infarcted myocardium at Day1, Day3 and Day7 post-AMI. Heart tissue homogenates were prepared and the following procedures were performed according to the manufacturer’s instructions.

### Apoptosis determination by flow cytometry

After pretreated with exosomes for 24 h, MSCs were exposed to hypoxia and serum deprivation (H/SD) conditions. An Annexin V-FITC/PI Kit (Becton, Dickinson and Company, USA) was utilized to evaluate the apoptotic MSCs according to the manufacturer’s protocols. Viable MSCs were defined as Annexin V^−^/PI^−^, early apoptotic MSCs as Annexin V^+^/PI^−^ and late apoptotic and necrotic MSCs as Annexin V^+^/PI^+^. The proportion of apoptotic MSCs was calculated after adding the number of early and late apoptotic cells together.

### Quantitative real-time polymerase chain reaction (qRT-PCR)

TRIzol reagent (Life Technologies, USA) was used to extract total RNA from MSCs following the manufacturer’s instructions. A Prime-Script^TM ^RT Reagent Kit with gDNA Eraser (Takara, Japan) was used to perform mRNA reverse transcription. Quantitative real-time PCR was conducted with PowerUp™ SYBR™ Green Master Mix (Applied Biosystems, USA) on a QuantStudio 3 Real-Time PCR system (Applied Biosystems, USA). The expression level of mRNA was normalized to β-actin, and the data were calculated via comparative 2^−ΔΔCt^ methods. mRNA primer sequences were synthesized by TianyBiotech and listed as follows: β-actin: Forward: 5′- CCCGCGAGTACAACCTTCTT-3′, Reverse: 5′-CGCAGCGATATCGTCATCCA-3′; CXCR4: Forward: 5′-TTCCTCGGGGCCAAATTCAA-3′, Reverse: 5′-GTGGAGACGGAAGAGTGTCC-3′. Each experiment included 3 technical replicates and at least 3 independent repeats.

### Western blotting

Exosomes and MSCs were collected and then lysed in RIPA lysis buffer (Thermo Fisher Scientific, USA) with a protease inhibitor cocktail (Roche, Germany), and protein concentrations were quantified by BCA protein assay (Beyotime, China). Proteins (20 μg) were loaded with 4× loading buffer, separated on a 4–12% Bis–Tris gel (Invitrogen, USA) and transferred onto PVDF membranes (Millipore, USA). Then, the membrane was blocked with 5% bovine serum albumin or skim milk in TBST for 2 h and incubated with primary antibodies against TSG101 (Santa Cruz Biotechnology, 1:1000, USA), Alix (Cell Signaling Technology, 1:1000, USA), CXCR4 (Proteintech, 1:1000, China) and β-actin (Cell Signaling Technology, 1:2000, USA) at 4 °C overnight. After incubated with the corresponding secondary antibodies (Beyotime, 1:3000, China) for 1 h at room temperature, protein bands were detected with chemiluminescence imaging system (Tanno-5800multi, China). The density of the target bands was normalized to that of β-actin.

### Statistical analysis

Statistical data are presented as the mean ± standard error of the mean (SEM) and were analyzed using GraphPad 8.0 (GraphPad Software, USA). Normal distribution was determined by Shapiro–Wilk test. The difference of normal variates was tested by Student’s *t* test within two groups. One-way ANOVA test followed by Tukey post hoc test (variance homogeneity) or Dunnett’s T3 test (variance non-homogeneity) was used for multiple comparisons (three or more groups). Non-normal data were analyzed by Mann–Whitney test or Kruskal–Wallis test with a Dunn post-test was used for multiple comparisons (three or more groups) with normally distributed variables. Statistical significance was set at *p* < 0.05 for all comparisons.

## Results

### Identification of exosomes derived from MSCs

**Fig. 1:**
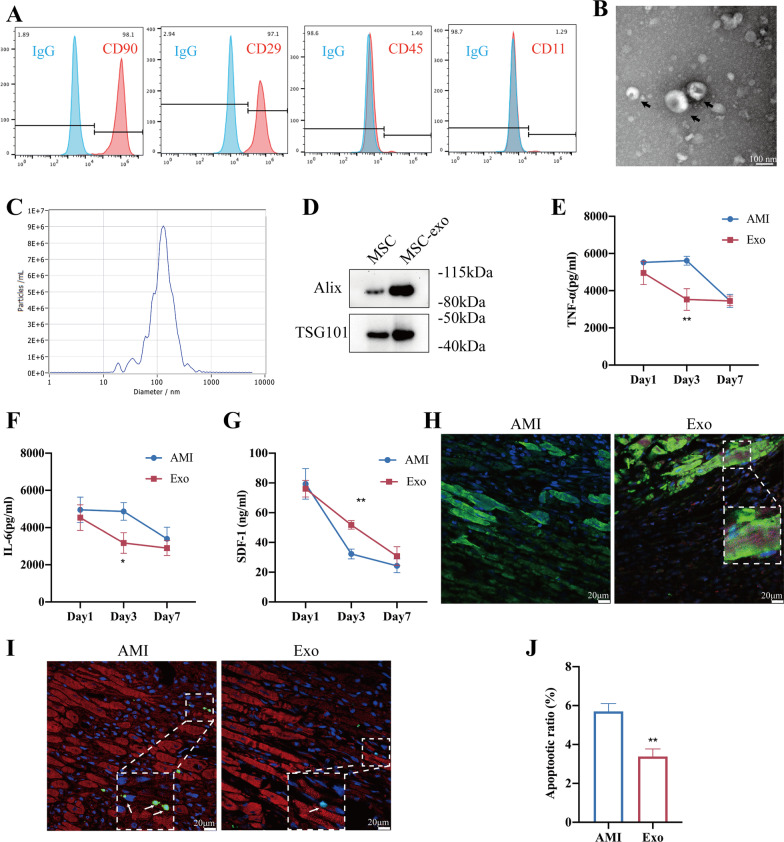
Characterization of exosomes derived from MSCs and their therapeutic effects. **A** Flow cytometry of the cell surface markers of MSCs. **B** Transmission electron microscopy was used to identify the shape of exosomes. Scale bar = 100 nm. **C** Nanoparticle tracking analysis was utilized to analyze the particle size of exosomes. **D** Detection of exosomal protein markers was performed using western blotting. Quantification of TNF-α (**E**) and IL-6 (**F**) levels in the peri-infarcted myocardium by ELISA (*n* = 6). **G** Quantification of SDF-1 levels on Day 1, Day 3 and Day 7 postinfarction (*n* = 5). **H** Distribution of PKH-26-labeled exosomes in the infarcted heart on Day 3 after AMI. Scale bar = 20 μm. Representative images (**I**) and quantification (**J**) of apoptotic cells in the peri-infarcted myocardium in the AMI or Exo group on Day3 (*n* = 4). All data are shown as the mean ± SEM. Student’s *t* test or one-way ANOVA followed by Tukey’s test was performed to assess statistical significance. *p < 0.05, **p < 0.01 versus AMI group

MSCs at passage 3 were typically adherent spindle-shaped cells and were identified by flow cytometry as exhibiting CD90^+^, CD29^+^, CD45^−^ and CD11^−^ expression (Fig. 1A). TEM and NTA were used to identify the shape and size of MSCs-derived exosomes, which were typically cup-shaped with an average size of 121.6 nm (Fig. 1B, C). Western blot analysis indicated that the isolated exosomes expressed exosome-specific proteins such as Alix and TSG101 (Fig. 1D).

### Exosome delivery reduced inflammation and apoptosis and elevated myocardial SDF-1 levels

We first injected PBS (100 μL) or PKH26-labeled exosomes (20 μg, in 100 μL PBS) into the peri-infarcted myocardium 30 min post-AMI. Exosome delivery significantly reduced inflammatory cytokines levels compared to levels in the AMI group, as indicated by the ELISA results for TNF-α and IL-6 (Fig. 1E, F). Consistent with previous reports [35, 36], the ELISA results showed that SDF-1 levels in peri-infarcted myocardium in the AMI group peaked at Day 1 postinfarction and then rapidly declined to a low level within the first week. Conversely, exosome delivery enhanced SDF-1 levels and maintained it at a relatively high level on Day 3, although it decreased to a low level on Day 7 (Fig. 1G). On Day 3 post-AMI, the confocal images indicated that PKH26-labeled exosomes were still retained in myocardium (Fig. 1H), and the exosome delivery significantly reduced the number of the apoptotic cells compared to that observed in the AMI group (Fig. 1I, J). To sum up, intramyocardial injection of exosomes resulted in decreased inflammation levels, elevated myocardial SDF-1 expression levels and reduced apoptosis in infarcted border zone on Day 3 postinfarction, making it an appropriate time for MSCs transplantation. Thus, we chose Day 3 postinfarction to deliver the MSCs to evaluate the effects of combined pretreatment in modulating the therapeutic efficacy of MSCs.

### Exosome and the combinatorially pretreated MSC delivery improved cardiac function postinfarction

**Fig. 2:**
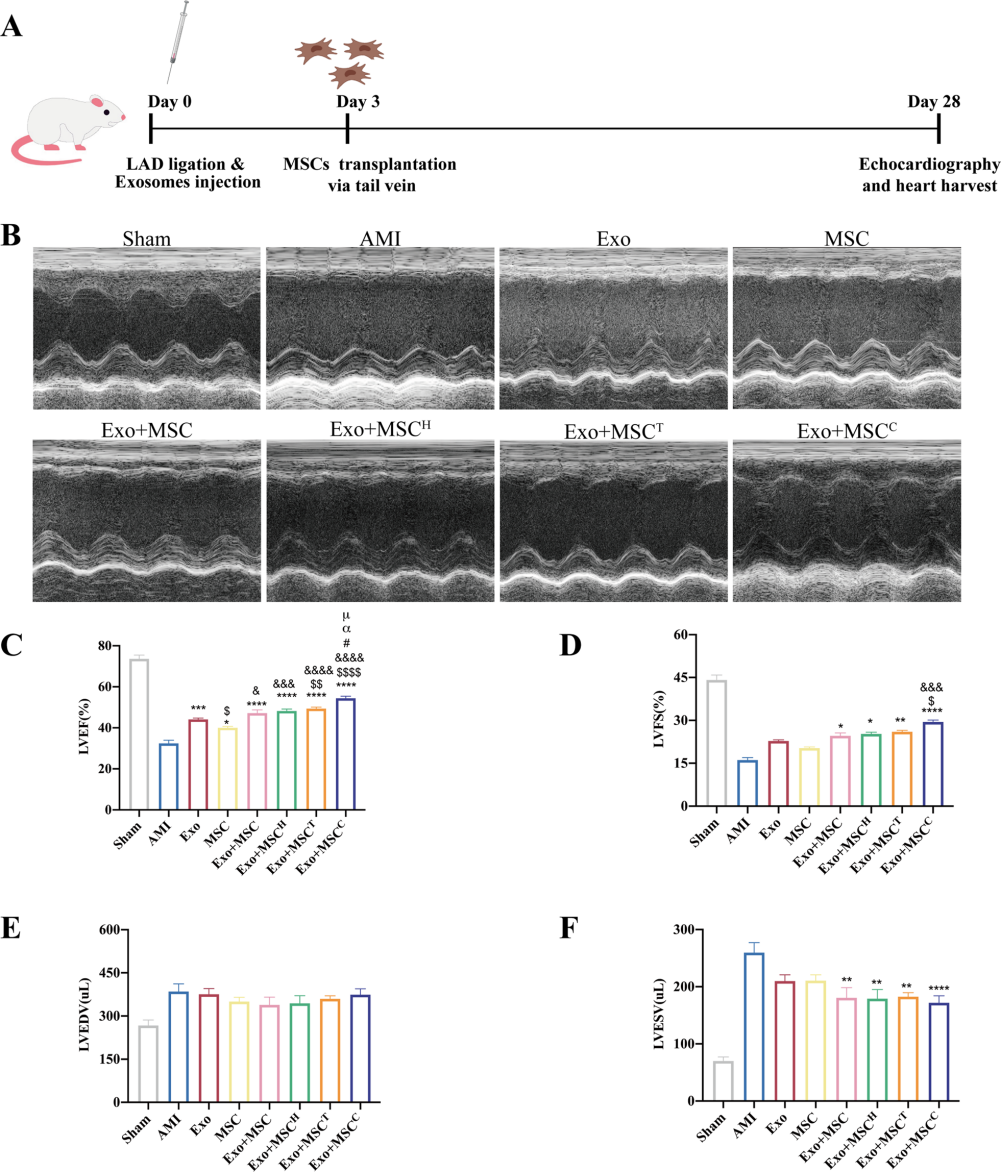
Transplantation of exosomes and the combinatorially pretreated MSCs improved cardiac function postinfarction. **A** Schematic diagram of transplantation of exosomes and differentially pretreated MSCs in a sequential manner. **B** Representative echocardiogram images of rat hearts 4 weeks after AMI. LVEF (**C**), LVFS (**D**), LVEDV (**E**) and LVESV (**F**) of different groups were calculated (*n* = 9–10 for each group). Statistical data are expressed as mean ± SEM and were analyzed using one-way ANOVA followed by Tukey’s. *p < 0.05, **p < 0.01, ***p < 0.001, ****p < 0.0001 versus AMI group; ^$^*p* < 0.05, ^$$^*p* < 0.01, ^$$$^*p* < 0.001, ^$$$$^*p* < 0.0001 versus Exo group; ^&^*p* < 0.05, ^&&^*p* < 0.01, ^&&&^*p* < 0.001, ^&&&&^*p* < 0.0001 versus MSC group; ^#^*p* < 0.05 versus Exo + MSC group; ^α^*p* < 0.05 versus Exo + MSC^H^ group; ^μ^*p* < 0.05 versus Exo + MSC^T^ group

To evaluate whether the combinatorially pretreated MSCs exerted better therapeutic efficacy, exosomes were first injected intramyocardially into the peri-infarcted hearts 30 min after injury, and MSCs (including MSC, MSC^H^, MSC^T^, or MSC^C^) were then injected through the tail vein at Day 3 post-AMI (Fig. 2A). Compared to the AMI group, the Exo group had significantly ameliorated the AMI-induced LV dilation on Day 28 and co-delivery therapy exerted better effects (Fig. 2B). Marked decreases in LVEF and LVFS were observed in the AMI group, indicating the deteriorated cardiac function. Sequential transplantation of exosomes and MSCs led to a significant elevation in LVEF and LVFS, and the Exo + MSC^C^ group showed the highest improvement in cardiac function among all cotransplantation groups (Fig. 2C–F).

### Exosomes and combinatorially pretreated MSCs transplantation dramatically ameliorated infarct size and collagen area

**Fig. 3:**
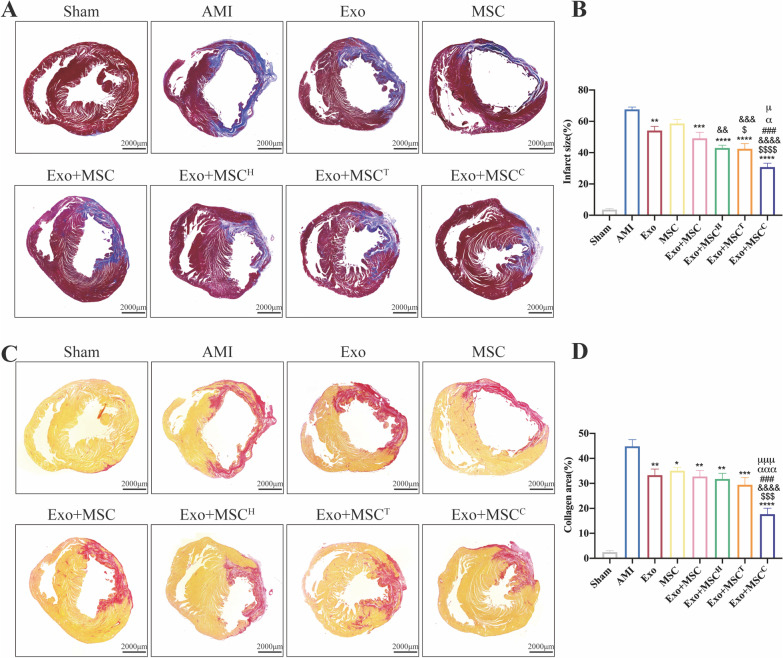
Transplantation of exosomes and the combinatorially pretreated MSCs reduced infarct size and ameliorated fibrosis after AMI. Representative Masson trichrome staining images (**A**) and quantitative analysis (**B**) of infarct size at 4 weeks post-AMI (*n* = 7), Scale bar = 2000 μm. Representative Sirius red staining images (**C**) and quantification (**D**) of the collagen area at 4 weeks post-AMI (*n* = 7), Scale bar = 2000 μm. Data are presented as mean ± SEM and were analyzed by one-way ANOVA followed by Tukey’s test. *p < 0.05, **p < 0.01, ***p < 0.001, ****p < 0.0001 versus AMI group; ^$^*p* < 0.05, ^$$^*p* < 0.01, ^$$$^*p* < 0.001, ^$$$$^*p* < 0.0001 versus Exo group; ^&^*p* < 0.05, ^&&^*p* < 0.01, ^&&&^*p* < 0.001, ^&&&&^*p* < 0.0001 versus MSC group; ^#^*p* < 0.05 versus Exo + MSC group; ^α^*p* < 0.05 versus Exo + MSC^H^ group; ^μ^*p* < 0.05 versus Exo + MSC^T^ group

Since exosomes and the combinatorially pretreated MSCs therapy achieved elevated cardiac function, we further evaluated the infarct size and collagen area. The Exo + MSC^H^, Exo + MSC^T^ and Exo + MSC^C^ groups achieved a reduced infarct size compared to Exo group at 4 weeks post-AMI. Among the three groups, the Exo + MSC^C^ group exhibited a lower infarct size compared to both the Exo + MSC^H^ and Exo + MSC^T^ groups (Fig. 3A, B). Similar to the infarct size results, the Exo + MSC^C^ group demonstrated the lowest collagen area compared with all the other three cotransplantation groups (Exo + MSC, Exo + MSC^H^ and Exo + MSC^T^) (Fig. 3C, D). Taken together, these results showed that sequential delivery of exosomes and the combinatorial pretreated MSCs led to a reduced infarct size and collagen area, while Exo + MSC^C^ might be the best transplantation strategy for the treatment of AMI.

### Exosomes and combinatorially pretreated MSCs delivery augmented angiogenesis after AMI

**Fig. 4:**
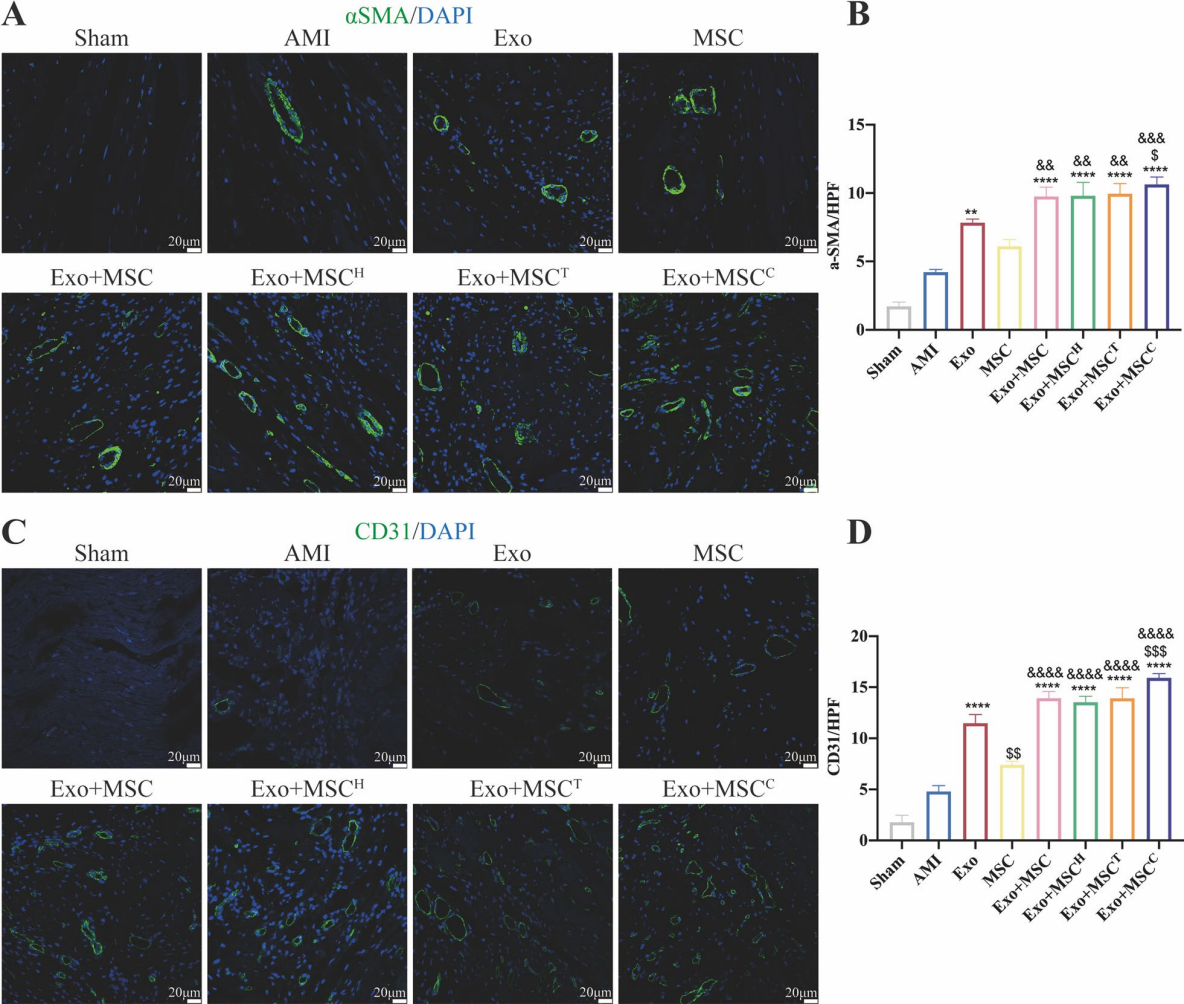
Sequential transplantation of exosomes and the combinatorially pretreated MSCs promoted angiogenesis in the infarcted hearts. Representative images of tissue sections of α-SMA (green) staining (**A**) and quantification (**B**) of α-SMA^+^ cells per HPF at 4 weeks post-AMI (*n* = 5). Scale bar = 20 μm. Representative images of CD31 (green) staining (**C**) and quantitative data (**D**) of CD31^+^ cells per HPF (*n* = 5), Scale bar = 20 μm. All data are presented as mean ± SEM and compared by one-way ANOVA with Tukey’s test. *p < 0.05, **p < 0.01, ***p < 0.001, ****p < 0.0001 versus AMI group; ^$^*p* < 0.05, ^$$^*p* < 0.01, ^$$$^*p* < 0.001 versus Exo group; ^&^*p* < 0.05, ^&&^*p* < 0.01, ^&&&^*p* < 0.001, ^&&&&^*p* < 0.0001 versus MSC group

To evaluate whether exosomes and the combinatorially pretreated MSCs delivery induces morphometric changes in the ischemic heart, we measured the arteriolar and capillary densities by quantification of α-SMA staining (Fig. 4A, B) and CD31 staining, respectively (Fig. 4C, D). Exosomes transplantation alone as well as all cotransplantation treatments achieved increased arteriolar and capillary density compared to that observed in the AMI group. Notably, Exo + MSC^C^ showed the highest arteriolar and capillary density among all combinatorial treatments.

### The combination of hypoxia and TXL pretreatment enhanced the effects of MSCs by increasing CXCR4 levels

**Fig. 5:**
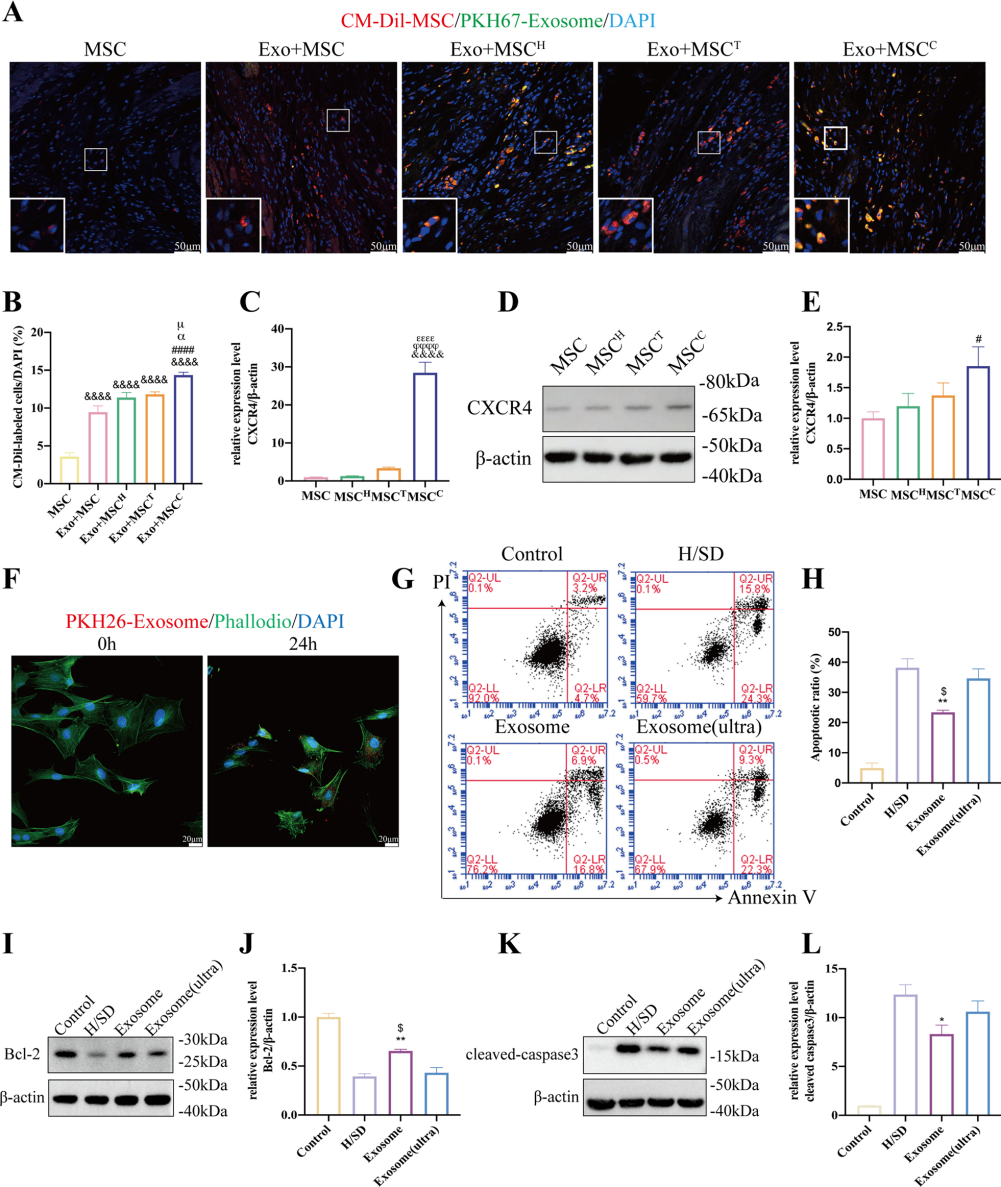
Combined pretreatment with hypoxia and TXL elevated the expression level of CXCR4 and increased the retention rate of transplanted MSCs. Combined pretreatment with hypoxia and TXL elevated the retention rate of the transplanted MSCs in infarcted myocardium (**A**, **B**) (*n* = 5). Scale bar = 50 μm. Combined pretreatment with hypoxia and TXL significantly elevated both the mRNA (*n* = 4) (**C**) and protein levels (*n* = 5) (**D**, **E**) of CXCR4 in MSCs. After 24 h of incubation, PKH-26-labeled exosomes could be taken up by MSCs (**F**). Under H/SD conditions, exosomes effectively protected MSCs against apoptosis (**G**, **H**) (*n* = 4). Representative images of western blotting (**I**, **K**) and quantitative data (**J**, **L**) of the protein levels of Bcl-2 and cleaved-caspase 3 (*n* = 4). Statistical data were analyzed using one-way ANOVA followed by Tukey’s test with data shown as mean ± SEM. ^&&^*p* < 0.05, ^&&^*p* < 0.01, ^&&&^*p* < 0.001, ^&&&&^*p* < 0.0001 versus MSC group; ^#^*p* < 0.05, ^##^*p* < 0.01, ^###^*p* < 0.001, ^####^*p* < 0.0001 versus Exo + MSC group; ^α^*p* < 0.05 versus Exo + MSC^H^ group; ^δϕϕϕϕ^*p* < 0.0001 versus MSC^H^ group; ^εεεε^*p* < 0.0001 versus MSC^T^ group; ^μ^*p* < 0.05 versus Exo + MSC^T^ group; *p < 0.05, **p < 0.01 versus H/SD group; ^$^*p* < 0.05 versus Exosome (ultra) group

To better elucidate the mechanisms underlying the superior cardioprotective effects observed for Exo + MSC^C^, we evaluated the retention rate of the engrafted MSCs in infarcted myocardium via fluorescent dye tracing. The CM-Dil-labeled MSCs were injected through tail vein at Day 3 after the delivery of PKH67-labeled exosomes. Combined delivery of exosome and MSCs showed higher percentage of CM-Dil^+^ cells compared to the single MSC group. And significantly more CM-Dil^+^ cells were found in the Exo + MSC^C^ group than in the other combinatorial groups, indicating a higher recruitment rate (Fig. 5A, B). Collectively, these results showed that the combined pretreatment with hypoxia and TXL increased the retention of transplanted MSCs. Since the SDF-1/CXCR4 axis is vital in the recruitment of MSCs to infarcted myocardium [49–51], we further investigated the effects of combinatorial pretreatment on CXCR4 levels in MSCs. Both the mRNA and protein levels of CXCR4 in MSCs were significantly elevated after combined pretreatment with hypoxia and TXL (Fig. 5C–E).

### Exosomes protected MSCs against hypoxia-induced apoptosis in vitro

To further determine the effect of pre-injection of exosomes on the transplanted MSCs, we pretreated MSCs with exosomes for 24 h. PKH-26-labeled exosomes could be taken up by MSCs after 24 h of incubation (Fig. 5F). As flow cytometry indicated, the Exo group had significantly reduced apoptosis of MSCs compared to the H/SD and H/SD + Exo (Ultra) groups, in which exosomes were ruptured by ultrasonication (Fig. 5G, H). In addition, Exo treatment markedly elevated the anti-apoptotic protein Bcl-2 level and decreased the pro-apoptotic cleaved-caspase 3 level of MSCs (Fig. 5I–L) under H/SD conditions. Collectively, these results demonstrated that exosomes efficiently protected MSCs against hypoxia-induced injury.

## Discussion

**Fig. 6:**
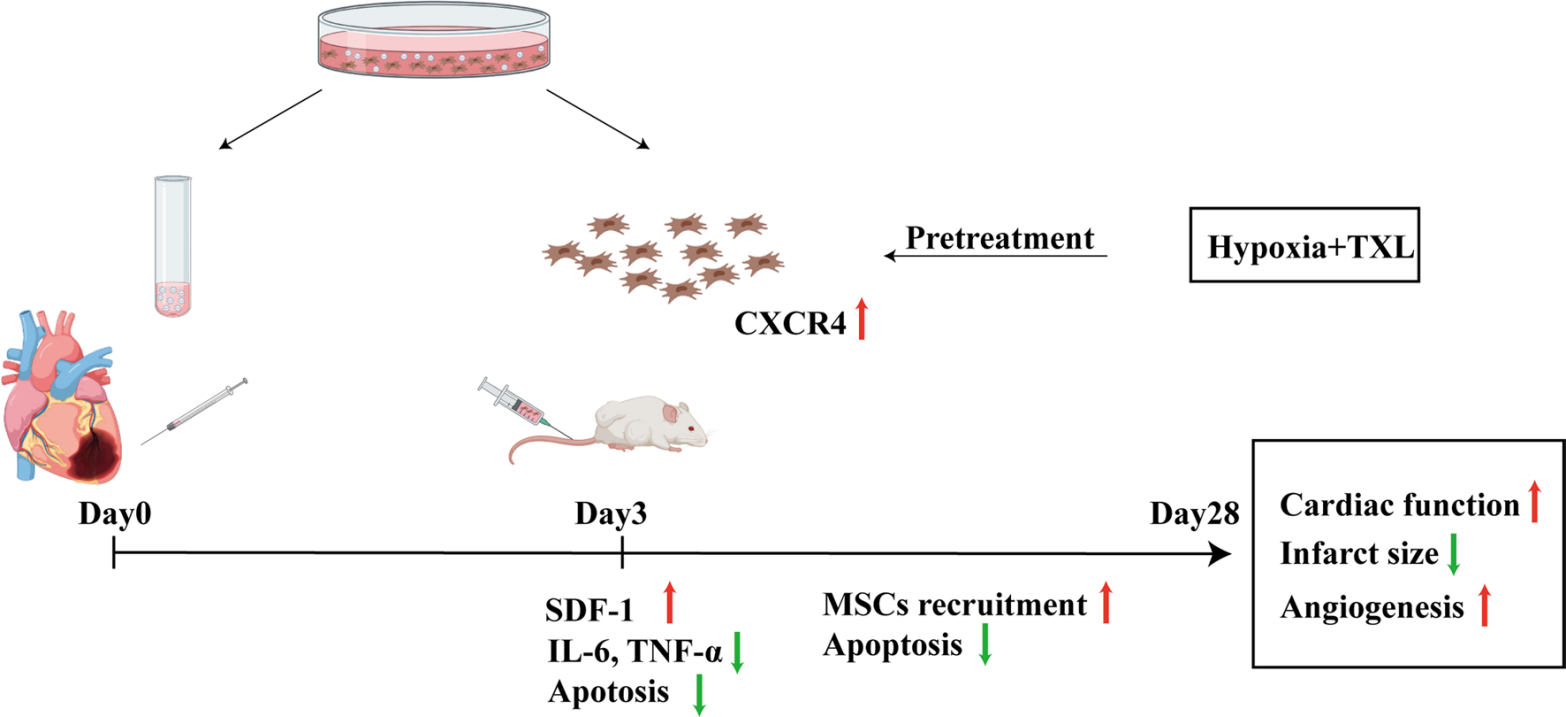
Effects and mechanisms of sequential transplantation of exosomes and the combinatorially pretreated MSCs in the treatment of AMI. Delivery of exosomes into infarcted myocardium 30 min after infarction markedly elevated myocardial SDF-1 levels, decreased inflammatory cytokines levels including TNF-α and IL-6 and reduced apoptosis at Day 3 post-AMI, ameliorating the harsh microenvironment which was beneficial for the recruitment and survival of the sequentially transplanted MSCs. In addition, combinatorial pretreatment of hypoxia and TXL augmented the CXCR4 level and increased the recruitment of transplanted MSCs to the infarcted myocardium. These effects ultimately led to significantly enhanced cardiac function, angiogenesis and reduced infarct size

Although various types of stem cells seem to be promising strategies for facilitating cardiac repair post-ischemic injury, the low retention rate and poor survival of engrafted cells have restricted their therapeutic efficacy in the treatment of AMI [12, 13]. Exosome transplantation have been demonstrated to ameliorate inflammation and preserve the cardiac function [28–32]. Therefore, we explored whether exosome delivery could ameliorate the harsh microenvironment to facilitate the recruitment and survival of stem cells, and we investigated whether combined pretreatment with hypoxia and TXL could better enhance the effects of the transplanted MSCs. The main findings were as follows: (1) Exosome delivery significantly augmented SDF-1 expression in peri-infarcted myocardium, with limited inflammation and reduced apoptosis on Day 3 post-AMI; (2) sequential delivery of MSCs after exosome injection significantly facilitated cardiac repair; and (3) combined pretreatment with hypoxia and TXL increased the CXCR4 level, which augmented the recruitment of transplanted MSCs and achieved the best therapeutic efficacy (Fig. 6).

As a promising cell-free therapy for promoting cardiac repair [52, 53], exosomes derived from MSCs have been found to have anti-apoptotic [28, 29, 31], anti-inflammatory [32, 54], as well as pro-angiogenic effects [55, 56], all of which are crucial to restore the function of infarcted myocardium. However, the short half-life and mild efficacy limited the therapeutic application of exosomes [53, 57]. In this study, we demonstrated that early injection of exosomes effectively ameliorated inflammation, reduced apoptosis and enhanced the expression level of SDF-1 at Day 3 post-AMI. In addition, exosome pretreatment effectively protected MSCs against hypoxic injury. Collectively, with reduced inflammation, apoptosis and increased SDF-1 levels, Day 3 post-AMI was an appropriate time for sequential transplantation of MSCs. Zhang et al. pretreated cardiac stem cells (CSCs) with exosomes derived from MSCs (MSC-Exo), and MSC-Exo-preconditioned CSCs significantly enhanced angiogenesis, reduced cardiac fibrosis and elevated cardiac function [58]. Shi et al. also reported that the pretreatment of CSCs with MSC-Exo enhanced the therapeutic effects in ischemic myocardium [59]. Therefore, exosomes derived from MSCs could be used as a new therapeutic vehicle for facilitating stem cell-mediated cardiac repair.

Due to the limited retention and survival rate of MSCs, various strategies have been developed to modify the therapeutic efficacy of MSCs including drug pretreatment [36, 37], hypoxia preconditioning [38, 39], genetic modification [40–42] and tissue engineering [43, 44, 60]. Of note, cell sheet transplantation has turned out to be a novel option in MI therapy, which possesses the advantages of prolonging retention and survival, improving engraftment and prognosis [60–63]. Cell sheets derived from modified MSCs transplantation might provide with novel insights into enhancing therapeutic effects in MI treatment. As reported, hypoxia pretreatment enhanced the expression level of CXCR4 and reduced the apoptosis under hypoxic injury [64–66]. In addition, drug pretreatment, including commonly clinically used TXL, has been demonstrated to protect MSCs against hypoxia-induced injury [45]. Therefore, we chose to combine hypoxia and TXL pretreatment to test whether the cardioprotective effects of MSCs could be enhanced. Four weeks post-AMI, MSCs pretreated with hypoxia and TXL achieved the highest retention rate and best performance in cardiac repair. Further analysis indicated that the combined hypoxia and TXL pretreatment significantly upregulated the expression levels of CXCR4 in MSCs. It is worth noting that SDF-1 and its receptor CXCR4 are pivotal in MSCs-mediated cardiac repair [49–51]. The level of SDF-1 in the peri-infarcted myocardium was immediately upregulated and rapidly declined, while the elevated SDF-1 level facilitated the recruitment of CXCR4^+^ cells [25]. CXCR4, as the key receptor involved in MSCs recruitment to the infarcted myocardium, is a chemotactic receptor recognizing the chemokine SDF-1. Overexpression of CXCR4 in MSCs resulted in decreased LV remodeling and enhanced LV function, indicating that modifying the CXCR4 expression level was beneficial for post-infarction myocardial repair [67]. Since exosome delivery elevated the myocardial level of SDF-1, the upregulated CXCR4 level obtained with the combined pretreatment was beneficial for the recruitment of transplanted MSCs to the injured myocardium. For patients with AMI, early injection of exosomes could ameliorate the harsh microenvironment and prolong the therapeutic window for MSC transplantation. Although further large animal experiments and clinical trials are needed, this sequential transplantation approach provides novel insights into the clinical treatment of AMI.

Stem cell therapy is a promising strategy for augmenting myocardial repair, but its clinical use is highly restricted by the low retention and poor survival of engrafted cells. In recent years, novel strategies, including MSCs modifications and combining cell therapy, have been developed to enhance the efficacy of stemcell-based therapy [68, 69]. In this study, we found that sequential transplantation of exosomes and combinatorially pretreated MSCs further facilitated cardiac recovery. Although the effects of cardiac repair remain to be examined in preclinical and clinical research, current advances have indicated the promising potential of stem cell therapy in facilitating cardiac repair post-ischemic injury. Considering the promising potential, continuous exploration to develop novel cell-based approaches, such as combining stem cells and exosomes with biomaterials, and combinational use of different stem cell types, might help to achieve better cardiac recovery.

## Conclusions

Transplantation of exosomes and the combined pretreated MSCs in a sequential manner effectively facilitated cardiac repair post-AMI. Transplantation of exosomes into ischemic hearts 30 min postinfarction markedly modulated the ischemic milieu, including reducing the levels of inflammatory TNF-α and IL-6 factors, elevating SDF-1 expression levels and promoting the survival of MSCs. The combined pretreatment with hypoxia and TXL effectively enhanced the expression level of CXCR4, thus achieving better performance in facilitating cardiac repair, which offers novel insights into stem cell-mediated cardiac repair.

## Data Availability

All data generated or analyzed during this study are included in this published article.

## Abbreviations

CVD: Cardiovascular disease
MI: Myocardial infarction
MSC: Mesenchymal stem cell
SDF-1: Stromal cell-derived factor 1
CXCR4: CXC chemokine receptor 4
TXL: Tongxinluo
SD: Sprague–Dawley
TEM: Transmission electron microscopy
NTA: Nanoparticle tracking analysis
LAD: Left anterior descending
LVEF: Left ventricular ejection fraction
LVFS: Left ventricular fractional shortening
LVEDV: Left ventricular end-diastolic volume
LVESV: Left ventricular end-systolic volume
cTNT: Cardiac troponin T
HPF: High-power fields
α-SMA: α-Smooth muscle actin
H/SD: Hypoxia and serum deprivation
qRT-PCR: Quantitative real-time polymerase chain reaction
SEM: Standard error of mean
LV: Left ventricle
CSC: Cardiac stem cells
